# Microbial Sources of Amyloid and Relevance to Amyloidogenesis and Alzheimer’s Disease (AD)

**DOI:** 10.4172/2161-0460.1000177

**Published:** 2015-01-15

**Authors:** Y Zhao, P Dua, WJ Lukiw

**Affiliations:** 1 LSU Neuroscience Center, Louisiana State University Health Sciences Center, 2020 Gravier Street, New Orleans LA 70112 USA; 2 Department of Cell Biology and Anatomy, Louisiana State University Health Sciences Center, 1901 Perdido Street, New Orleans LA 70112 USA; 3 Department of Health Information Management, Louisiana State University Ruston LA 71270 USA; 4 Department of Ophthalmology, Louisiana State University Health Sciences Center, 533 Bolivar Street, New Orleans LA 70112 USA; 5 Department of Neurology, Louisiana State University Health Sciences Center, 1542 Tulane Avenue, New Orleans LA 70112 USA

**Keywords:** Alzheimer’s disease (AD), Beta amyloid precursor protein (βAPP), Congo Red (CR), Curli fibers, Hologenome, Inflammation, Innate-immunity, Toll receptor type 2 (TLR2), Senile (amyloid) plaques (SP)

## Abstract

Since the inception of the human microbiome project (HMP) by the US National Institutes of Health (NIH) in 2007 there has been a keen resurgence in our recognition of the human microbiome and its contribution to development, immunity, neurophysiology, metabolic and nutritive support to central nervous system (CNS) health and disease. What is not generally appreciated is that (i) the ~10^14^ microbial cells that comprise the human microbiome outnumber human host cells by approximately one hundred-to-one; (ii) together the microbial genes of the microbiome outnumber human host genes by about one hundred-and-fifty to one; (iii) collectively these microbes constitute the largest ‘diffuse organ system’ in the human body, more metabolically active than the liver; strongly influencing host nutritive-, innate-immune, neuroinflammatory-, neuromodulatory- and neurotransmission-functions; and (iv) that these microbes actively secrete highly complex, immunogenic mixtures of lipopolysaccharide (LPS) and amyloid from their outer membranes into their immediate environment. While secreted LPS and amyloids are generally quite soluble as monomers over time they form into highly insoluble fibrous protein aggregates that are implicated in the progressive degenerative neuropathology of several common, age-related disorders of the human CNS including Alzheimer’s disease (AD). This general commentary-perspective paper will highlight some recent findings on microbial-derived secreted LPS and amyloids and the potential contribution of these neurotoxic and proinflammatory microbial exudates to age-related inflammatory amyloidogenesis and neurodegeneration, with specific reference to AD wherever possible.

## Secretory Elements of the Human Microbiome

Humans contain a complex and dynamic community of microbes called ‘the microbiome’ that forms a ‘metaorganism’ with commensal or symbiotic benefit to the host [1–7]. Collectively these microbes constitute the largest ‘diffuse organ system’ in the body, more metabolically active than the liver, and through systemic effects impact the health and well-being of the human host [2–10]. The microbiome of the human gastrointestinal (GI) tract, containing the largest reservoir of microbes in the body, is composed of about 10^14^ microbes from at least 1000 distinct microbial species [2–6]. Interestingly, (i) microbial cells outnumber human host cells by about one hundred-to-one and microbial genes collectively outnumber human genes by about one hundred-and-fifty-to-one [2–4,11,12]; (ii) the vast majority of GI tract microbiota are anaerobic or facultative anaerobic bacteria, with fungi, protozoa, archaebacteria and other microorganisms making up the remainder [2–5]; (iii) of all mammalian species human GI tract microbiota with bacterial densities of up to 10^12^ per ml are the highest recorded density of any known microbial ecosystem [3–5]; and (iv) only two bacterial divisions (of the 52 divisions currently identified by metagenomics analysis) are prominent in GI tract microbiota, and these include the Gram-positive Firmicutes (~51%) and anaerobic Gram-negative Bacteroidetes (~48%). The remaining 1% of phylotypes are distributed amongst the Cyanobacteria, Fusobacteria, Proteobacteria, Spirochaetes and Verrucomicrobia, along with various species of fungi, protozoa, viruses and other commensal microorganisms [4,11–14]. That the Firmicutes and Bacteroidetes were preferentially selected from the 52 bacterial divisions available in the biosphere is of evolutionary interest with implications for the ‘hologenome’ theory, which postulates that it is not the individual organism, but rather the organism together with its associated symbiotic microbial communities that should be considered as the basic unit of natural selection and evolution [15]. Interestingly, and from what is known, microorganisms that make up the smallest 1% of the microbiome have a disproportionately large impact on, and relevance to, human disease, and a major function of the healthy GI tract microbiome is to keep the proliferation of any potentially pathogenic microbes under homeostatic control [5,12,16]. Indeed, besides being of immense benefit to human health and welfare in the extraction of energy from food, absorption of nutrients and generation of vitamins (such as vitamin B12 and K), amino acids (such as lysine) and peptide sugars (such as peptidoglycans), the microbiome anchors a systemic immune-defense system against infective pathogens, while simultaneously being a prodigious producer of bacterial LPS and amyloid. Here we discuss relatively recent research at the intersection of LPS, microbial and AD amyloid highlighting 6 recent, specific and highly illustrative insights into the potential contribution of microbial-derived LPS and amyloid to CNS amyloidosis and the degenerative disease process.

### Microbiome-Derived Amyloid

A remarkable variety of microbial species including bacteria and fungi of the human microbiome generate significant quantities of functional amyloid, and even early scientific interpretations suggested that secreted amyloid and other shed molecules may serve some immune-evasive and survival strategy [3,4,17]. From the 10^14^ microbiota of the human microbiome it is also apparent that humans tolerate a substantial exposure to LPSs and microbial-generated amyloid, which potentially may contribute to the pathology of progressive CNS diseases with an amyloidogenic component [3,4,18–20]. For example, amyloids are associated with structures located on fungal surfaces and the recent observation of amyloidogenic fungal proteins and diffuse mycoses in the blood of AD patients suggest that chronic fungal infection contributes to AD risk via amyloid exposure [2–6,21,22]. To cite another recent example, in Escherichia coli *(strain K12)* extracellular bacterial amyloids known as ‘curli fibers’ and composed of the major structural curli subunit gA (CsgA) are a common secretory component used as structural materials facilitating surface attachment and adhesion, biofilm development and protection against host defenses [23,24]. Curli fibers typically engulf and surround bacteria forming a meshwork or ‘biofilm’ that biophysically connects large numbers of bacteria together [18,19]. Biofilms therefore represent a matrix of extracellular polymeric amyloids and other complex lipoproteins and LPS in various structural forms. Interestingly, the extracellular ~18 kDa CsgA amyloid precursor contains a pathogen-associated molecular pattern (PAMP) that, like the Aβ42 peptide, is recognized by the human immune system toll-like receptor 2 (TLR2; see below) [25,26]. An expanding list of bacterial amyloid systems include those associated with gram-negative species of Bacillus, Pseudomonas, Staphylococcus, Streptomyces and others, suggesting that functional amyloids are a widespread phenomenon extensively generated by a wide range of microbiome bacteria and recognized by TLR2 [3,4,23,24,27]. Indeed the extremely large number and variety of microbiome bacteria and their capability to produce enormous quantities of LPS, amyloid and LPS/amyloid breakdown products indicates that human physiology may be chronically exposed to a tremendous systemic amyloid burden of a wide variety of amyloid, and this may be especially important during the course of aging when both the GI tract epithelium and blood-brain barriers become significantly more restructured and permeable [2,27–30].

### The Amyloid Peptides of Alzheimer’s Disease (AD)

‘Amyloid’ is a generic term for any aggregated, insoluble, lipoprotein-rich deposit exhibiting β-pleated sheet structures that are oriented perpendicular to the fibrillar axis [31–34]. About 55% of all eukaryotic proteins are predicted to contain unstructured regions of amino acids that are intrinsically amyloidogenic [34–36]. The amyloids that characterize AD consist largely of perivascular amyloid enriched in the 40 amino acid Aβ40 peptide, parenchymal amyloid, enriched in the 42 amino acid Aβ42 peptide, and nuclear amyloids that contain highly complex mixtures of lipoprotein fibrils and amyloid aggregates [34,37,38]. The Aβ peptides of AD are derived from a polytopic transmembrane β-amyloid precursor protein (βAPP) though tandem beta- and gamma-secretase cleavage events [39–41]. Cellular trafficking of the ~770 amino acid βAPP precursor is regulated by a large βAPP interactome that includes membrane integral and membrane peripheral adaptor proteins, and also by interactions with membrane-associated glycolipids and phospholipids [34,42]. Aβ40 peptides associate with endothelial cells that line the cerebral vasculature, and the more neurotoxic, albeit less abundant, hydrophobic Aβ42 peptides form the central core of the senile plaque (SP) of the parenchymal lesions that characterize AD [40,43]. The extra two hydrophobic amino acids in the Aβ42 peptide appear to convey many of the neurotoxic biophysical properties and self-aggregation of this slightly larger molecule [25,26,44,45]. The recognition of Aβ42 peptides and their misfolded aggregates by microglial surveillance systems, and the inability of microglial cells to deal with these toxic, pro-inflammatory inclusions, especially in their multimeric and aggregated form are thought to form the molecular basis for the aberrant immune activation, chronic inflammation and elevated oxidative stress that is characteristic of AD neuropathology [8,34,39,46–51]. Notably, (i) Aβ42 peptides as monomers, dimers and fibrils induce patterns of inflammatory gene expression typical of the classical innate-immune and inflammatory response induced by infectious agents such as bacterial LPS, a common endotoxin of the outer membrane of gram-negative bacteria [49,52; see below]; (ii) the presence of bacterial LPS or endotoxin-mediated inflammation strongly contributes to amyloid neurotoxicity [19,24–27,32,42,50–55]; and (iii) AD amyloids, like prion amyloids, once formed, may induce a self-perpetuating process leading to amplification, aggregation and spreading of pathological protein assemblies, and serial propagation of distinct strains of Aβ prion-like amyloids from AD patients have been recently observed [56,57]. Indeed, an increasing number of studies support the idea (i) that certain self-propagating protein conformations feature in the pathogenesis of several common neurodegenerative diseases including AD; (ii) that pro-inflammatory and immunogenic aggregates of Aβ peptides may become self-propagating in AD brain; and (iii) that certain forms of Aβ peptides are serially transmissible and hence important in the propagation of neurological disease [57]. The contribution of LPS to the serial transmissibility of certain amyloidogenic Aβ peptides and their aggregates is currently not well understood, however it has recently been shown that Aβ peptide fibrillogenesis is strongly potentiated by soluble bacterial endotoxins, suggesting the contribution of infectious events and/or microbial-released factors to the pathogenesis of AD [24,54,57].

### Bacterial LPS and PAMPS

LPS, sometimes referred to as bacterial lipoglycan or bacterial endotoxin, is the major amphiphilic glucosamine-based phospholipid of the outer membrane of Gram-negative bacteria, and in non-encapsulated bacteria are directly exposed to their environment [19,58–60]. LPS consists of a hydrophobic and lipophilic inner core, a hydrophilic outer core polysaccharide chain, and a repeating hydrophillic O-antigenic oligosaccharide chain (N~4–40) specific to the bacterial serotype [19,61]. LPSs are initially soluble but over time LPSs typically form large heterogeneous aggregates with molecular masses of 1–4 Mda or greater, and these structures are exceptionally immunogenic to the host [61–65]. Activation via LPS involves interactions with innate immunity-receptors such as Toll-like receptor 4 (TLR4; see below) in complex with MD-2 protein (also known as lymphocyte antigen 96) and accessory proteins, such as the cluster of differentiation innate-immune protein 14 (CD14) and LPS-binding protein [19,63,64]. LPS and MD-2 specifically activate the human TLR4 leading to the production of a highly pleiotropic mixture of cytokines and chemokines which in turn promote inflammatory and innate-immune responses [61–64]. Bacterial LPS is considered to possess a prototypical pathogen-associated molecular pattern or PAMP, consisting of LPSs arranged in a highly specific molecular configuration efficiently recognized by host innate-immunity [60,65]. Recent literature has provided evidence that LPS is involved in the inflammatory and pathological processes associated with Aβ peptide-mediated amyloidosis characteristic of AD. For example it has been demonstrated just this year: (i) that the chronic infusion of LPS into the fourth ventricle of rats reproduces many of the inflammatory and pathological features seen in the AD suggesting that bacterial LPS potentiates the fibrillogenesis of Aβ peptides [24]; (ii) that LPS-induced neuroinflammation is associated with AD-type amyloidogenic axonal pathology and dendritic degeneration in rodent models of AD [59]; and (iii) that the glycosylphosphatidyl-inositol-anchored LPS and ‘microbe-detecting receptor CD14, crucial in the neutralization of invading microbes, is also stimulated by Aβ fibrils thus further linking innate-immune signaling with AD amyloidogenesis [60,64]. This later observation of a CD14-dependent inflammatory response to Aβ fibrils indicates a structural molecular mimicry between the highly hydrophobic, aggregated Aβ fibrils and biophysically similar microbial PAMPS that contribute to chronic pro-inflammatory signaling and progressive neural degeneration [59,60,65,66]. Such findings again underscore the pathogenic importance of both microbial-released factors and the Aβ fibrils that characterize AD-type neuropathology [3,4,24,66].

### Amyloidophilic Dyes: Congo Red Stains Microbial LPS and Amyloid

The water soluble secondary diazo dye Congo Red [CR; 3,3'-([1,1'-biphenyl]-4,4'-diyl)bis(4-aminonaphthalene-1-sulfonic acid-disodium salt)] has been classically used in microbiological epidemiology and investigative microbiology as a bacterial stain due to its important and unusual spectroscopic properties, and high affinity for both LPS and amyloid [67–69]. For example, CR rapidly identifies the presence of virulent forms of the gram-negative Proteobacteria Shigella where the dye binds unique surface LPS repeat structures of this facultative anaerobe [33,67,68]. CR’s apple-green birefringent fluorescence enhancement under polarized light is probably due to (i) a substrate-mediated hydrophobic pi-pi orbital stacking interaction between the aromatic rings of CR dye molecules and β-pleated sheet or related structures of both LPS and amyloids [69] and (ii) a restriction of the torsional rotation of the CR molecule upon binding [3,4,69]. The polymerization of amyloidogenic proteins, such as AD and prion amyloids into ordered β-pleated sheet, or similar structures, is cooperative, and can be accelerated by already aggregated amyloid in a highly selective ‘seeding’ process. Indeed the CR-based intercalation of β-pleated sheets, induction of a positive anisotropy that is directionally dependent and polarized, and generation of a measureable wavelength shift and apple-green birefringence is a hallmark of the majority of both microbial and AD amyloid [33,68]. The identification of the ‘amylome’, a classification of amino acid sequences within proteins with internal, self-complementary interfaces and high fiber-forming capability has improved our understanding of the tendency of different proteins to form amyloids that contribute to ‘dense-deposit’ disease [34,40,70]. Still widely used, CR staining is a highly sensitive diagnostic tool for amyloidosis and the ‘gold standard’ for the detection of amyloid fibrils including the Aβ42-enriched perivascular, parenchymal and nuclear amyloid deposits of AD [33,38,67,68]. Interestingly, it has very recently been demonstrated: (i) that the index of accumulation of CR-positive nuclear amyloids in spherical nucleoplasmic microenvironments may predict brain cell fitness and survival [38]; (ii) that LPS is capable of inducing a more pathogenic CR-sensitive β-pleated sheet conformation of prion amyloids [55]; and (iii) that the infectious microbial burden is significantly associated with both AD development and the propensity of AD amyloids to be stained by CR [66,70].

### Molecular Mimicry

Molecular mimicry is defined (i) as the hypothetical possibility that structural similarities in amino acid sequence or PAMPS between microbial and host molecules, including amyloids, are sufficient enough to result in the cross-activation of the immune system leading to autoimmunity, and progressively pathological, pro-inflammatory signaling; and (ii) may exemplify one of the most powerful strategies that prokaryotic pathogens and eukaryotic parasites utilize to override homeostatic host cell functions to ensure their own replication and survival [3,4,58]. For example, mitochondria appear to have originated from archaeobacteria via endosymbiotic relationships that formed very early in eukaryotic evolutionary history, and cross-reactivity of mitochondria and immunological responses to bacterial LPS or amyloids may exert deleterious auto-immune effects and drive dysfunction to mitochondrial homeostasis. These potentially devastating auto-immune effects may be exacerbated over the course of aging when both GI-tract and blood brain barriers of the CNS become more permeable and critical CNS components become ‘decompartmentalized’[2,28–30,58]. This is partially exemplified by the extragastric diseases such as basal ganglia disorder Sydenham’s chorea, rheumatic fever, low grade systemic inflammatory states and the link to the Firmicute Streptococcus and/or the gram-negative microaerophilic Proteobacteria Helicobacter pylori [3,4,9,16,71,72]. Previous bacterial infection resulting in antibody formation to amyloids or bacterial endotoxins may predispose CNS mitochondria or amyloids to subsequent attack by antibodies and immune effects that result in an up-regulation of CNS inflammation [58,66].

### Activation of TLR2 by Amyloids

Toll-like receptors (TLRs) constitute a family of 13 (TLR1 to TLR13) currently identified, non-catalytic type I membrane-spanning protein receptors expressed in macrophages and in dendritic and microglial cells. As pattern recognition receptors, TLRs play key roles in the innate-immune surveillance system by sensing structurally conserved PAMPs (see above) on microbial surfaces that are distinguishable from the host organism, and hence serve as immune-sensors that are a first line dense against microbial invasion [73–75]. Microglial TLR2s are activated by amyloid, bacterial lipoproteins, LPS and other microbial triggers that subsequently induce cytokine production, inflammation, phagocytosis and innate-immune defense responses that directly impact CNS homoeostasis and drive neuropathology. More specifically the TLR2/TLR1 complex can recognize biofilm-associated amyloids produced by Firmicutes, Bacteroidetes, and Proteobacteria [2,24,76]. Interestingly, (i) Aβ42 peptides that associate with microglia-mediated pro-inflammatory host responses also co-activate TLR2 signaling [74,77]; (ii) microbial amyloids further induce pro-inflammatory interleukin IL-17A, a driver of NF-kB signaling and cyclooxygenase-2 activation; (iii) LPS and amyloids also induce other potent mediators of inflammatory responses such as IL-22 that direct TLR2 activation [76]; and (iv) pathologically up-regulated levels of IL-17A and IL-22 are associated with chronic inflammatory diseases including AD [41,45].

## Concluding Remarks

Considerable advancement in DNA and RNA sequencing and bioinformatics technologies have created a new arena of human genetic research called ‘metagenomics’ that permits the comprehensive examination of complex microbial ecosystems without the need for labor-intensive microbial culture. This ‘metagenomic’ approach allows analysis of genetic material harvested directly from the GI tract microbiome using prokaryotic ribosomal RNA as an internalized reference without the need for microbial culture – indeed many microbial species of the human GI tract cannot be cultured ex vivo [6,13]. Currently the HMP is focused on the creation of the first integrated dataset of biological properties from both the microbiome and host from cohort studies of diseases with which the microbiome is associated [18,20,78]. The integration of complementary microbiome genus-and-species and abundance analysis using control microbial reference strains in cognitively normal aging humans is providing unprecedented information about the complexity of human-endogenous microbial communities and their potential contribution to CNS health and disease [6,13]. It is reasonable to speculate that the vast contribution and bioavailability of both microbial nutrients and neurotoxins are altered as humans age, leading to disequilibrium of the ‘hologenome’ that in part defines the difference between healthy aging from disease. Interestingly, at the precise time-point of death the human microbiome rapidly transforms into the ‘thanato-microbiome’ (thanatos-, Greek, death) and begins to play a primary role in the decomposition of host tissues; so keeping that potentially pathogenic fraction of the 10^14^ microbial species of the human GI tract in check, and the preservation of a healthy and homeostatic microbiome is an important part of the maintenance and preservation of human life itself [13,79]. Clearly, not only individual microbial abundance in the microbiome is important but also microbial speciation and stoichiometry, and what microbial complexities might contribute to, or detract from, optimum CNS health.

**Figure 1:**
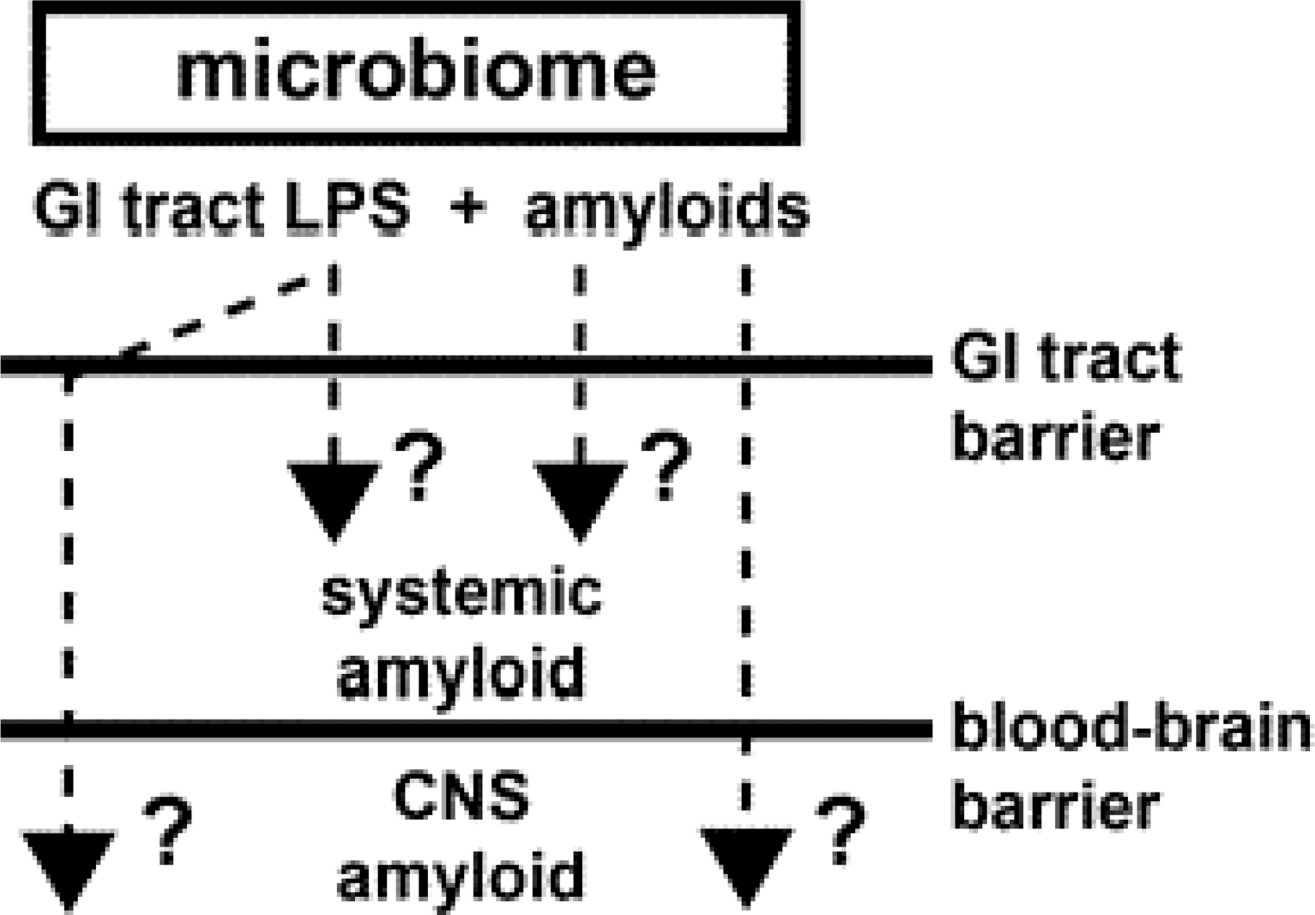
Potential contribution of gastrointestinal (GI) tract microbiome-derived lipopolysaccharide (LPS) and amyloids to systemic and central nervous system (CNS) amyloid burden

In summary a remarkable variety of microbes which constitute the human microbiome are capable in generating vast quantities of LPS and functional amyloid. The ability of both microbial- and CNS-derived amyloids to bind CR has provided a practical tool for characterizing their abundance, subcellular location and biophysical properties and has suggested a potential overlap in their occurrence in CNS disease [25,26,50]. Indeed microbial and CNS amyloids are very similar in their biophysical and biological structure, immunogenicity and ability to be stained with diazo dyes such as CR and complex mechanistic interrelationships between these amyloids and with LPS are beginning to emerge. The large amount microbial-generated LPS and GI tract amyloid implicates high potential for human systemic exposure to these microbial-sourced amyloids, especially since the bioavailability of amyloid to the CNS may increase as humans age when GI-tract and blood brain barriers of the CNS become more permeable [2,28,29,30]. What is noteworthy is that (i) that human microbes that produce amyloids such as CsgA and curli, and the Aβ42 peptides that accumulate in AD, are recognized by the same TLR2/TLR1 immune sensor-receptor system of the 13 different TLR-type receptors available; (ii) that all of these amyloids similarly direct increases in IL-17A- and IL-22-mediated pro-inflammatory signaling; and (iii) that Aβ42 peptides and CsgA do not share any common sequences of amino-acid, only considerable structural similarity in their PAMPs [80,81]. Microbes and their secretory exudates that include LPSs and amyloids are powerful pro-inflammatory and innate-immune activators and inducers of complement proteins and cytokines, that subsequently affect vascular permeability, immunogenicity and the generation of free radicals that further intensifies amyloid aggregation and inflammatory degeneration highly characteristic of age-related AD neuropathology, that includes defective Aβ peptide clearance mechanisms [14,82,83]. A more complete understanding of the human ‘hologenome’, of human microbial ecosystems and their secretory products should yield further insight into their contribution to age-related neurological diseases associated with amyloidogenesis, CNS inflammation and progressive neurodegeneration. It would be certainly informative to establish: (i) if LPSs, amyloids or other microbial-derived factors generated by the microbiome become more available systemically as humans age; (ii) if any microbial-generated amyloids or related signaling molecules co-localize with the amyloid-dense SP deposits or other insoluble lesions that characterize AD; (iii) what the evolution and nature of amyloid-related communication between the microbiome and the CNS has on the development or propagation of amyloidogenesis throughout the CNS; (iv) how these highly interactive factors impact the onset, propagation and course of age-related inflammatory neurodegeneration; and (v) how increased understanding of microbiome-mediated mechanisms of amyloidogenesis may lead to the advancement of more effective anti-amyloid therapeutic strategies (Figure 1).

This highly schematicized drawing shows that as a major part of the microbiome, GI tract microbial sources of LPS and/or amyloid may contribute to both systemic amyloid and CNS amyloid burden and impact amyloidogenesis in both systemic and CNS compartments (question marks). This may be particularly relevant during the course of aging when the GI tract barrier and blood-brain barriers become considerably more permeable. Such mechanisms may operate both directly via LPS/amyloid leakage through compromised GI tract or blood-brain barriers and/or indirectly through LPS/amyloid-triggered cytokines or other small pro-inflammatory molecules which transit normally protective physiological barriers. Indeed, microbes and their secretory exudates are extremely powerful pro-inflammatory and innate-immune activators and inducers of complement proteins and cytokines, that subsequently affect vascular permeability, immunogenicity and the generation of free radicals. These further promote amyloid aggregation and inflammatory degeneration characteristic of an age-related AD neuropathological processes that also involve defective Aβ peptide clearance mechanisms [14,81–83]. See text for further details.
